# Transformative Potential of Induced Pluripotent Stem Cells in Congenital Heart Disease Research and Treatment

**DOI:** 10.3390/children12060669

**Published:** 2025-05-23

**Authors:** Mohammed A. Mashali, Isabelle Deschênes, Nancy S. Saad

**Affiliations:** 1 Department of Physiology and Cell Biology, Dorothy M. Davis Heart and Lung Research Institute, College of Medicine, The Ohio State University Wexner Medical Center, Columbus, OH 43210, USA; isabelle.deschenes@osumc.edu; 2 Department of Surgery, Faculty of Veterinary Medicine, Damanhour University, Damanhour 22514, Egypt; 3 Department of Pharmacology and Toxicology, Faculty of Pharmacy, Helwan University, Cairo 11795, Egypt

**Keywords:** congenital heart disease, induced pluripotent stem cells, disease modeling, precision medicine, CRISPR/Cas9, regenerative therapies, genetic mutations

## Abstract

Congenital heart disease (CHD), the most common congenital anomaly, remains a significant lifelong burden despite advancements in medical and surgical interventions. Induced pluripotent stem cells (iPSCs) have emerged as a groundbreaking platform in CHD research, offering patient-specific models to investigate the genetic, epigenetic, and molecular mechanisms driving the disease. Utilizing technologies such as CRISPR/Cas9 gene editing, cardiac organoids, and high-throughput screening, iPSCs enable innovative strategies in disease modeling, precision drug discovery, and regenerative therapies. However, clinical translation faces challenges related to immaturity, differentiation variability, large-scale feasibility, and tumorigenicity. Addressing these barriers will require standardized protocols, bioengineering solutions, and interdisciplinary collaboration. This review examines the critical role of iPSCs in advancing CHD research and care, demonstrating their potential to revolutionize treatment through patient-specific, regenerative approaches. By addressing current limitations and advancing iPSC technology, the field is positioned to pave the way for precision-based CHD therapies for this lifelong condition.

## 1. Introduction

Congenital heart disease (CHD) represents a significant challenge to global healthcare, as the most common congenital anomaly [1] and as a leading contributor to childhood morbidity and mortality [2,3]. Despite substantial progress in surgical and medical interventions, CHD often necessitates lifelong care, leaving survivors vulnerable to complications that persist into adulthood. This burden emphasizes the need for transformative approaches that address the underlying causes of CHD rather than solely mitigating its structural and symptomatic effects [4].

**Figure 1:**
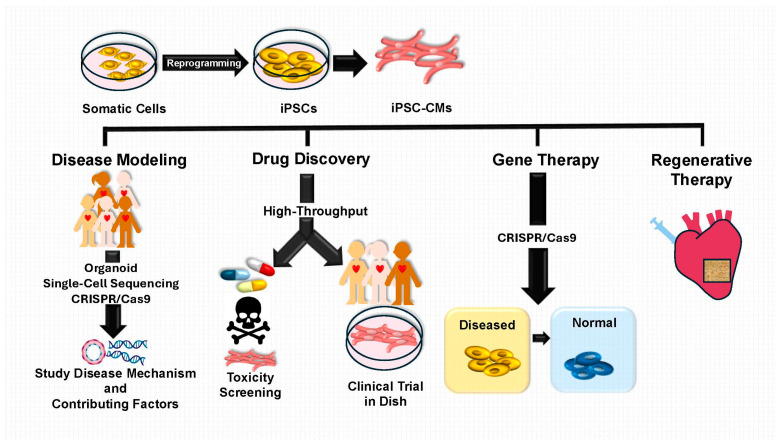
Induced pluripotent stem cell generation and applications in congenital heart disease research. CRISPR/Cas9, clustered regularly interspaced short palindromic repeats/CRISPR-associated protein 9; iPSCs, induced pluripotent stem cells; iPSC-CMs, iPSC-derived cardiomyocytes.

The discovery of induced pluripotent stem cells (iPSCs) has revolutionized the landscape of biomedical research, providing an innovative platform to investigate and address complex conditions such as CHD [5,6]. First introduced in 2006 by Takahashi and Yamanaka, iPSCs have revolutionized the field [7]. These cells are generated by reprogramming mature somatic cells into a pluripotent state via key transcription factors (OCT4, SOX2, KLF4, c-MYC) [7,8]. This groundbreaking approach overcomes many ethical and practical limitations associated with embryonic stem cells [9,10], while retaining the remarkable ability to generate diverse cell types. From cardiomyocytes to endothelial and smooth muscle cells, iPSCs offer an unparalleled tool for replicating the complexity of human biology, firmly establishing themselves as a key innovation in disease modeling, drug discovery, and regenerative medicine [11]. Figure 1 illustrates the process of iPSC generation and its applications in CHD research.

A critical advantage of iPSCs lies in their ability to create patient-specific models that capture the unique genetic and phenotypic characteristics of individuals [12,13]. This capability is particularly relevant for CHD, which arises from a complex interplay of genetic, epigenetic, and environmental factors [14,15,16]. By creating controlled environments to study genetic mutations, signaling pathways, and cellular interactions, iPSC-based models provide crucial insights into disease mechanisms and support the development of targeted therapies [17,18,19,20,21,22,23].

Moreover, iPSCs facilitate precision medicine by enabling personalized drug testing and cell replacement therapies without the need for immunosuppression [24]. iPSC-derived cardiomyocytes (iPSC-CMs) have facilitated high-throughput drug screening to assess both therapeutic efficacy and cardiotoxicity in CHD research [25,26]. Advances in tissue engineering using iPSCs, such as the development of cardiac patches and valves, offer new opportunities for regenerative therapies to repair structural heart defects [27].

Despite these promising advancements, several challenges remain, including the immaturity of iPSC-derived cells, variability in differentiation, and large-scale feasibility, all of which hinder their clinical application. However, innovations in bioengineering, 3D culture systems, and co-culture techniques are steadily addressing these barriers. This review explores how iPSCs are bridging critical gaps in disease modeling, drug discovery, and regenerative therapies for CHD. It emphasizes the importance of translational applications and interdisciplinary collaboration in accelerating the development of precision-based treatments.

## 2. iPSC-Based Modeling of CHD

### 2.1. Advances in iPSC-Based Disease Modeling

Traditionally, CHD research has relied on animal models, including genetically engineered mice, to study cardiac development and disease mechanisms. While these models have provided essential insights, they often fail to accurately replicate human CHD phenotypes due to differences in genomic content, physiology, and disease progression [28]. For instance, orthologous genetic variants in mice may not produce the same clinical manifestations observed in humans. These limitations underscore the need for human-based platforms that better reflect the complexities of CHD [28].

iPSCs offer an advanced solution by providing human-derived cells capable of replicating key aspects of cardiac development and disease progression (Figure 1). Unlike animal models, iPSCs can be differentiated into patient-specific cardiomyocytes, enabling researchers to study both genetic and epigenetic factors that influence CHD. For example, studies have shown that epigenetic memory can enhance the efficiency of cardiac differentiation, as demonstrated by differences in promoter methylation of transcription factors such as NKX2-5 and GATA4 between fibroblast-derived and cardiac progenitor cell (CPC)-derived iPSCs [29]. Such findings illustrate how iPSCs can uncover critical regulatory mechanisms and support the development of targeted interventions.

**Figure 2:**
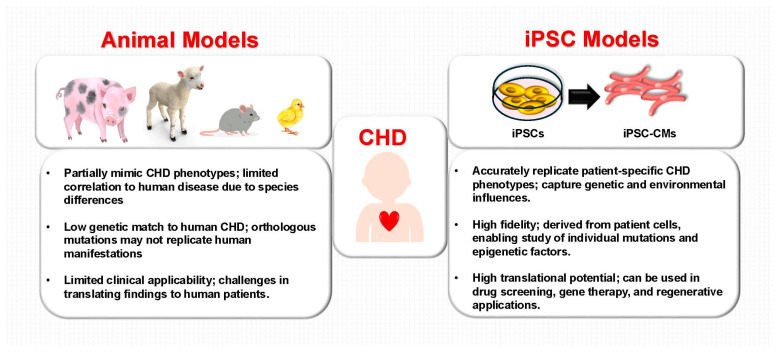
Limitations of animal models and the advantages of induced pluripotent stem cells in congenital heart disease research. Unlike animal models, iPSCs more accurately capture patient-specific characteristics, enabling precise disease modeling and the development of individualized therapies. CHD, congenital heart disease; iPSCs, induced pluripotent stem cells; iPSC-CMs, iPSC-derived cardiomyocytes.

By overcoming the limitations of animal models, iPSCs provide a robust foundation for translational research, enabling scientists to better evaluate therapeutic strategies in human-relevant contexts (Figure 2).

### 2.2. Patient-Specific iPSC Models for CHD

iPSCs derived from CHD patients enable the modeling of specific subtypes, including hypoplastic left heart syndrome (HLHS) [22,30,31,32], Tetralogy of Fallot (TOF) [17,33], pulmonary atresia with intact ventricular septum (PAIVS) [34,35], bicuspid aortic valve (BAV) and calcific aortic valve disease (CAVD) [36], supravalvular aortic stenosis (SVAS) [37], cardiac septal defects [38], and Barth syndrome (BTHS) [39] (Table 1). iPSC models have revealed how defects in key pathways, such as NOTCH signaling, contribute to abnormal cardiac morphogenesis in HLHS [22,23,31,32,40]. For example, an HLHS patient-specific iPSC line carrying a heterozygous

NOTCH1 mutation has been established and characterized, offering a valuable platform for dissecting disease mechanisms and testing therapeutics [32]. Similarly, iPSC-CMs from TOF patients have shown altered expression of genes implicated in collagen pathways, such as BICC1 and MYH11, which are linked to disease pathology [17]. These patient-specific models allow researchers to analyze disease mechanisms on an individualized basis, supporting the development of precision therapies tailored to specific genetic mutations and phenotypes (Figure 1).

### 2.3. iPSC-Derived Cardiac Organoids

Organoids are three-dimensional (3D) self-organizing structures [41] that replicate mature tissue architecture [42] and are often described as models that closely resemble the composition of adult tissues [43,44]. In cardiac systems, however, organoids typically represent embryonic and immature stages derived through the recapitulation of developmental programs [45,46,47]. Despite this immaturity, cardiac organoids derived from iPSCs mark a significant advancement in disease modeling by replicating the architecture and functionality of native cardiac tissue, offering a more physiologically relevant model than traditional two-dimensional cultures [48]. iPSC-derived organoids enable the study of complex disease phenotypes, such as structural abnormalities and functional defects in CHD [49].

For example, studies using TBX5 knockout cardiac organoids have demonstrated that this transcription factor plays a critical role in first heart field development and chamber formation [50]. These models revealed defects ranging from delayed morphogenesis to the absence of chamber structures [51,52]. These defects are accompanied by disruptions in electrophysiological and contractile functions, closely mimicking congenital heart defects such as atrial and ventricular septal defects. Similarly, Tbx1 knockout organoids have demonstrated significant disruption in second heart field development, resulting in impaired anterior heart tube elongation [53] and outflow tract (OFT) malformations [54,55,56], as seen in conditions like DiGeorge syndrome.

Additionally, organoid models have been employed to investigate the effects of environmental factors, such as pharmaceutical agents, on cardiac development. Research has shown that drugs like thalidomide and doxorubicin can disrupt myocardial development [57] and electrophysiological activity [47,58]. Thalidomide downregulates critical cardiac genes like Nppa and Vegf, leading to abnormal morphogenesis [59], while doxorubicin induces apoptosis and impairs contractile function [58], mimicking tissue damage observed in clinical cases of congenital defects.

### 2.4. Single-Cell Technologies in iPSC-Based CHD Research

Single-cell technologies, such as single-cell RNA sequencing (scRNA-seq), have significantly advanced the understanding of iPSC-derived cardiac cells [60,61] by revealing their heterogeneity [62] and offering insights into cellular mechanisms underlying CHD [63]. For example, scRNA-seq analysis of iPSC-CMs from patients with PAIVS identified distinct subpopulations with downregulated contractile and maturation genes alongside upregulated immature isoforms. These findings provide crucial insights into disrupted signaling pathways and transcription factors like HOPX and PDLIM3, which contribute to right ventricular hypoplasia and contractile dysfunction [64].

Similarly, transcriptomic profiling of iPSC-CMs derived from CHD patients, including those with single ventricle defects (SVD) and TOF, has uncovered significant gene expression differences compared to non-CHD controls. For example, 919 differentially expressed genes (DEGs) in SVD-iPSC-CMs were linked to developmental processes, while TOF-iPSC-CMs exhibited 250 DEGs with enrichment in developmental pathways [18]. These findings emphasize the importance of single-cell analysis in revealing how genetic mutations impact cardiac cell differentiation and function. Integrating single-cell and spatial transcriptomics with patient-specific iPSC models and organoid technologies provides a robust framework for mapping cellular heterogeneity and gene expression gradients during cardiac development [65,66]. This approach is invaluable for uncovering molecular mechanisms that drive CHD progression and guiding the development of targeted therapies [67,68]. Building upon advancements in modeling, iPSCs also offer unparalleled insights into the genetic and molecular underpinnings of CHD, enabling targeted therapeutic development.

**Table 1:** Induced pluripotent stem cell-based models in congenital heart disease.

CHD Subtype	iPSC-Based Model	Key Findings	Genetic Pathways
HLHS	iPSC-CMs, cardiac organoids, endothelial cells	Altered calcium handling, mitochondrial dysfunction [69,70], dysregulated histone acetylation patterns that impaired differentiation [71,72,73], endothelial-mesenchymal transition defects [23]	NKX2-5, NOTCH1 [22,23,40], Myh6 [19]
TOF	Patient-derived iPSC-CMs, CRISPR-engineered TOF models	Dysregulated collagen expression [17], contractile defects, abnormal right ventricular development, disrupted metabolic pathways (butanoate metabolism) [74,75].	GATA4, TBX1, JAG1 [17]
BAV	iPSC-derived endothelial and smooth muscle cells	Abnormal valvulogenesis, endothelial dysfunction, early calcification	GATA4 [76,77], NOTCH1 [36]
SVAS	iPSC-derived vascular smooth muscle cells	Elastin deficiency leading to vascular abnormalities and stenosis [37]	ELN [37]
Cardiac Septal Defects ASD, VSD, AVSD	Patient-specific iPSC-CMs, 3D cardiac tissue models	Defective septal development, impaired myocardial proliferation, disrupted signaling pathways [38]	TBX5, GATA4 [78], NKX2-5 [38,78,79,80]
BTHS	iPSC-CMs with TAZ mutations	Mitochondrial dysfunction, impaired cardiolipin remodeling, excessive ROS generation [39]	TAZ, PPAR pathways [39]
LQTS	iPSC-CMs carrying patient-specific KCNQ1, KCNH2 mutations	Prolonged action potential duration, abnormal ion channel activity	KCNQ1, KCNH2, SCN5A [81,82,83]
LVNC	iPSC-CMs, Fibroblasts	Decreased ventricular development, deep trabeculae, metabolic maturation defects [84]	Mkl2, Myh7, Nkx2-5 [84]
HOS	iPSC-CMs	Epigenetic alterations affecting cardiac developmental genes [85]	TBX5 [86]
OFT malformations	iPSC-CMs, organoid [54,55,56]	Decreased transcription levels in cardiomyocytes	GATA6 [87]

Abbreviations: ASD, atrial septal defect; AVSD, atrioventricular septal defect; BAV, bicuspid aortic valve; BTHS, Barth syndrome; CHD, congenital heart disease; CRISPR, clustered regularly interspaced short palindromic repeats; HLHS, hypoplastic left heart syndrome; HOS, Holt–Oram syndrome; iPSC, induced pluripotent stem cell; iPSC-CMs, iPSC-derived cardiomyocytes; LQTS, long QT syndrome; LVNC, left ventricular non-compaction; OFT, outflow tract; PPAR, peroxisome proliferator-activated receptor; ROS, reactive oxygen species; SVAS, supravalvular aortic stenosis; TAZ, Tafazzin; TOF, tetralogy of Fallot; VSD, ventricular septal defect.

## 3. Molecular Insights and Precision Medicine Using iPSCs

### 3.1. Genetic and Epigenetic Contributions in iPSC Models

iPSC models have significantly advanced the understanding of CHD by elucidating the roles of genetic mutations in cardiac development. Mutations in key transcription factors such as GATA4, NKX2-5, and TBX5, which regulate cardiac development, have been linked to defects such as atrial and ventricular septal defects and TOF [88,89]. One notable study demonstrated that GATA4 mutations associated with atrial septal defects (ASDs) disrupt transcriptional networks responsible for atrial specification, impairing key signaling pathways such as Sonic Hedgehog and altering TBX5 recruitment at cardiac enhancers [38,79]. Likewise, iPSCs from TOF patients with TBX1 mutations highlighted defects in CPC proliferation, offering critical insights into OFT malformations [17]. Additionally, mutations in GATA6, a master regulator of heart formation, have also been linked to diverse congenital heart defects, including both cardiac malformations and pancreatic agenesis [90], underscoring its pivotal role during embryogenesis [91,92].

Recent research has also highlighted the role of de novo mutations in chromatin regulation genes, revealing genetic overlap between CHD and neurodevelopmental disorders [93,94,95]. In parallel, iPSC models have proven instrumental in exploring genotype–phenotype discordance and incomplete penetrance in inherited cardiac disorders. For example, a study using iPSC-CMs from individuals within the same LQTS Type 2 (LQT2) family revealed striking differences in cellular electrophysiology despite identical KCNH2 mutations. This approach led to the identification of genetic modifiers, such as REM2 and KCNK17, that explained disease variability and underscored the power of iPSC modeling in dissecting complex inheritance patterns [96].

In addition to genetic alterations, epigenetic mechanisms, including histone modifications, DNA methylation, and non-coding RNAs, play critical roles in regulating cardiac gene expression [97]. While genetic mutations provide foundational insights, epigenetic factors add complexity, shaping gene expression and cellular behavior. iPSC models have provided a platform for studying how dysregulated epigenetic states contribute to CHD [98]. For example, studies of HLHS-derived iPSCs revealed dysregulated histone acetylation patterns that impaired cardiomyocyte differentiation [71,72,73]. Furthermore, non-coding RNAs, including microRNAs (miRNAs) and long non-coding RNAs, also play crucial roles in cardiac development [99]. iPSC-CMs have identified 96 miRNAs that promote cardiomyocyte proliferation, many of which act on the Hippo signaling pathway by regulating components such as LATS2, TEAD1, and YAP, thereby highlighting their critical roles in cardiac biology and development and contributing to our understanding of CHD pathogenesis [100].

Transcriptomic analyses of iPSC-CMs have also uncovered distinct RNA profiles, shedding light on disrupted pathways regulating cardiomyocyte function and development. These findings highlight the significance of RNA-based regulatory mechanisms and their contribution to CHD pathogenesis, promoting further exploration of epigenetic influences [18]. Continued advancements in next-generation sequencing technologies are uncovering additional genetic variations, such as single-nucleotide polymorphisms and copy number variants, which further expand the potential for developing targeted therapies [101].

### 3.2. Patient-Specific Therapies Enabled by iPSCs

#### 3.2.1. Drug Testing and Screening with iPSC-CMs

iPSC-CMs have become invaluable tools for drug discovery and personalized treatment development. Large-scale studies, such as the Comprehensive In Vitro Proarrhythmia Assessment initiative, have demonstrated the ability of iPSC-CMs in predicting drug-induced arrhythmias across multiple laboratories [102,103]. These studies validate the utility of iPSC-CMs for assessing arrhythmogenic potential and modeling patient-specific responses. For instance, iPSC-CMs from patients with trastuzumab-induced cardiotoxicity have revealed underlying metabolic disruptions, enabling the identification of potential therapeutic targets [104,105]. Similarly, studies using iPSCs derived from various cardiovascular and neurological conditions have demonstrated their capacity to restore calcium signaling and contractile function through targeted drug interventions [106].

In the context of CHD and inherited cardiac disorders, iPSC-CMs have been instrumental in testing drugs for conditions such as long QT syndrome (LQTS) and catecholaminergic polymorphic ventricular tachycardia, successfully recapitulating disease phenotypes to evaluate antiarrhythmic therapies [81,82,83]. Patient-specific iPSC models of LQTS have provided key insights into the electrophysiological abnormalities associated with various LQTS subtypes and have facilitated precision medicine approaches by enabling high-throughput drug testing and genotype-specific treatment strategies [107]. Furthermore, research using HLHS-derived iPSCs has uncovered significant cellular defects, including altered calcium handling and mitochondrial dysfunction. Metabolic interventions targeting these defects have demonstrated improved contractility, suggesting potential therapeutic avenues [69,70]. iPSC-based studies on TOF have identified disrupted metabolic pathways, such as butanoate metabolism, further emphasizing the potential of metabolic modulators as therapeutic strategies [74,75].

Importantly, because CHD primarily affects pediatric populations, there is growing interest in utilizing iPSC-based platforms to assess pediatric-specific drug responses, developmental toxicity, and long-term safety profiles in children [21]. These models are especially valuable for younger patients with complex CHD subtypes who require tailored pharmacological strategies. Cardiomyocytes derived from pediatric patient-specific iPSCs offer unique advantages by capturing age-dependent electrophysiological and metabolic characteristics, enabling developmentally relevant modeling of drug efficacy and toxicity [108]. For example, iPSC-CMs from patients with HLHS have demonstrated mitochondrial and contractile dysfunctions, provided mechanistic insights, and revealed potential therapeutic targets [21]. Pediatric-focused iPSC models thus hold great promise for optimizing treatment outcomes and minimizing adverse effects in vulnerable pediatric populations, aligning with the goals of precision medicine in CHD care [108].

#### 3.2.2. Gene Therapy and Genome Editing Using iPSC Models

The combination of iPSC technology with CRISPR/Cas9 gene editing holds great promise for correcting disease-causing mutations [109], as shown earlier in Figure 1. One example involved the correction of a GATA4 mutation in iPSCs derived from ASD patients, resulting in restored cardiomyocyte differentiation and function [78]. This study exemplifies the potential of gene-edited iPSCs for personalized regenerative therapies aimed at repairing damaged cardiac tissue. Furthermore, preclinical studies have demonstrated that transplantation of gene-edited iPSC-CMs can improve cardiac function in animal models of heart disease [110,111]. For example, a meta-analysis highlighted functional recovery in ischemic heart disease models following iPSC-CM transplantation [111]. Research involving large animal models has further confirmed the feasibility of using iPSC-CMs for myocardial repair, paving the way for clinical applications [110]. 

In addition to therapeutic applications, CRISPR/Cas9 has advanced functional genomics research by enabling precise genetic modifications in iPSC models. This allows researchers to investigate the role of specific mutations in cardiac development [112,113]. For example, TBX5 knockout studies have elucidated the gene’s role in Holt–Oram syndrome (HOS) [114], while the correction of TBX5 mutations restored normal gene function and enhanced understanding of regulatory pathways [86]. Moreover, CRISPR technology facilitates the exploration of complex genetic contributions to CHD, including oligogenic inheritance [84]. By introducing or correcting combinations of genetic variants in iPSCs, researchers can study how multiple genetic factors interact to influence disease progression [115,116]. This capability enhances our understanding of CHD’s genetic complexity and supports the development of targeted therapies [117].

#### 3.2.3. Clinical Trials and Regenerative Applications of iPSC-Based Therapies

The first-in-human phase I/II clinical trial (LAPiS) demonstrated the feasibility of using iPSC-derived heart muscle cells injected directly into a patient’s myocardium during coronary bypass surgery. This trial marked a significant milestone in regenerative medicine for heart failure treatment [118]. Building on these advancements, a clinical trial by the Mayo Clinic investigates the potential of autologous iPSC-derived cardiac cells to strengthen heart function in CHD patients. In this study, a patient’s own skin cells are reprogrammed into iPSCs, differentiated into cardiac cells, and then delivered into the heart muscle to assess their therapeutic potential. This pioneering initiative exemplifies how iPSC-based therapies are transitioning from laboratory research to clinical practice [119].

Successful clinical translation of iPSC-based therapies will require alignment with regulatory frameworks, such as those outlined by the U.S. Food and Drug Administration (FDA) for cell-based products [120]. This includes preclinical safety evaluation, standardization of manufacturing under current good manufacturing practices (cGMP), and phase I/II clinical trial designs to assess safety, dosing, and preliminary efficacy [121]. Furthermore, partnerships with industry collaborators, including biotechnology companies and translational research institutes, are critical to scaling production, navigating regulatory approval, and advancing therapies toward clinical implementation [122].

## 4. iPSC-Based Drug Discovery and Testing for CHD

iPSC-derived models have revolutionized the drug discovery process, providing platforms for testing therapeutic efficacy and cardiotoxicity in patient-specific contexts. Recent advancements have enabled high-throughput screening to accelerate drug development for CHD.

### 4.1. High-Throughput Screening Platforms Using iPSC-CMs

**Figure 3:**
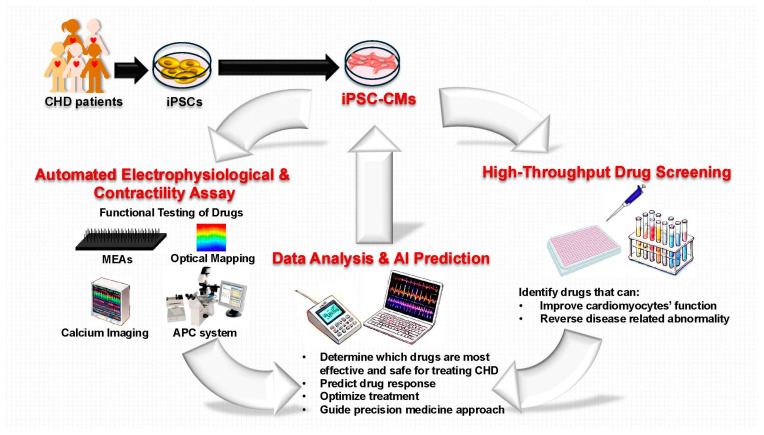
Induced pluripotent stem cell platforms for high-throughput drug screening in congenital heart disease. APC, automated patch clamp; CHD, congenital heart disease; iPSCs, induced pluripotent stem cells; iPSC-CMs, iPSC-derived cardiomyocytes; MEAs, microelectrode arrays.

iPSC-CMs serve as powerful tools for high-throughput drug screening, allowing researchers to evaluate compounds for their effects on contractility, calcium signaling, and electrophysiology (Figure 3). Studies using iPSC-CMs have identified drugs with both therapeutic potential and cardiotoxic risk [123]. 

To further enhance drug discovery efforts, advanced systems for generating and analyzing iPSC-CMs have significantly improved drug safety testing and disease modeling. Automated patch clamp (APC) systems have become a cornerstone of modern electrophysiological drug screening, offering high-throughput and highly precise measurements of ion channel activity and action potentials. When integrated with traditional techniques such as microelectrode arrays (MEAs), calcium imaging, and optical mapping, these platforms enable comprehensive, high-capacity assessments of cardiac electrophysiology and drug-induced effects [124]. High-throughput functional analysis of iPSC-CMs enables rapid and efficient screening of drug responses, accelerating the identification of effective treatments for CHD [125]. Complementing these efforts, innovations in automated physiological recording techniques, such as video motion tracking and optical mapping, have enabled precise, quantitative assessments of physiological defects in iPSC-derived cardiac cells carrying CHD-associated mutations. These technologies have contributed to the creation of scalable, high-throughput platforms that are instrumental in advancing drug discovery and personalized therapies [70,126].

Adding to these advancements, cardiac microphysiological systems (MPS) using hiPSC-CMs represent a cutting-edge approach to human-specific cardiac modeling. By creating 3D-aligned cardiac tissues with physiologically relevant properties, MPS platforms enable robust cardiotoxicity testing and high-throughput drug screening. This innovative technology bridges the gap between in vitro and in vivo models, offering more accurate predictions of human cardiac responses to therapeutic compounds [127]. These high-throughput platforms not only accelerate the identification of effective treatments but also provide a critical foundation for functional restoration studies, where compounds are tested for their ability to rescue impaired cardiac function in patient-specific models.

### 4.2. Functional Restoration Studies in iPSC Models

Phenotypic screening using iPSC-CMs allows for the identification of compounds that restore normal cardiac function in patient-specific models (Figure 3). This approach is particularly useful for investigating drugs that target impaired contractility and calcium handling, which are often disrupted in CHD. Among the tools used in this context, APC systems are especially valuable for quantifying how candidate compounds reverse abnormal electrophysiological properties in patient-specific iPSC-CMs. This level of precision facilitates the identification of drugs that restore normal cardiac function by correcting mutation-induced defects in ion channel behavior [124,128,129]. For instance, iPSC-CMs with CHD-associated mutations have helped identify small molecules capable of rescuing these functional defects, paving the way for targeted therapeutic interventions [5,6]. By focusing on patient-derived cells, researchers gain insights into the impact of specific genetic mutations on drug responses, enabling more effective and personalized treatments [108,130]. 

### 4.3. Technical Advances in iPSC-CM Differentiation and Scale-Up

To enable disease modeling and drug screening applications, several well-established protocols have been developed to convert iPSCs into iPSC-CMs. A widely adopted approach involves temporal modulation of Wnt/β-catenin signaling using small molecules: activation during mesoderm induction (e.g., CHIR99021), followed by inhibition (e.g., IWP2 or Wnt-C59) to promote cardiac specification. This monolayer-based protocol enables efficient and reproducible differentiation and has been adapted for multiple disease models [131]. For scalable production, bioengineering strategies such as suspension culture in spinner flasks, microcarrier-based expansion, and 3D cardiac organoids have been implemented to increase yield and enhance structural and functional maturation of iPSC-CMs [132,133,134]. Moreover, automation platforms and cGMP-compliant protocols are being developed to support high-throughput screening and therapeutic applications [133]. These technical innovations are critical for advancing iPSC-based CHD models toward translational and clinical utility.

### 4.4. Drug Repurposing Using iPSC-Derived Models

iPSC-based models have provided unique opportunities to repurpose existing drugs for CHD treatment. For example, statins, traditionally used for lipid regulation, have shown potential benefits in modulating cardiac remodeling in iPSC-derived CHD models. This strategy accelerates the translation of therapies into clinical practice, providing timely interventions for CHD [135].

## 5. Limitations and Challenges of iPSCs in CHD Research

Despite the transformative potential of iPSC technology, several limitations hinder its clinical application. These challenges necessitate innovative solutions and ongoing research to improve the reliability of iPSC-derived models.

### 5.1. Immaturity of iPSC-CMs

Human iPSC-CMs exhibit fetal-like characteristics, with disorganized myofibril structures and a lack of T-tubules, which contrast with the mature architecture of adult cardiomyocytes [136]. Efforts to promote maturation, such as the use of hormones [137], physical and electrical conditioning [138], and co-culture systems [139], have shown promise but remain limited in replicating adult cardiac phenotypes. Additionally, while 3D cardiac organoids provide a more physiologically relevant model [140], their ability to fully capture CHD pathogenesis remains uncertain.

### 5.2. Large-Scale Feasibility and Reproducibility

The large-scale production of iPSC-derived cells with consistent quality is a significant obstacle. Variability in reprogramming techniques, differentiation protocols, and genetic backgrounds often leads to inconsistent outcomes [141,142]. Addressing this requires standardized protocols, bioengineering innovations, and automation platforms, which can improve reproducibility [143,144]. Techniques discussed earlier, such as advanced high-throughput screening systems, are paving the way toward more efficient large-scale applications [125].

Several major collaborative initiatives are actively working to overcome variability and enhance reproducibility in iPSC research. For example, the National Institutes of Health (NIH)’s iPSC initiatives aim to develop standardized protocols for cell generation and differentiation to promote consistency across institutions [145]. The International Stem Cell Initiative (ISCI) provides a global framework for assessing the genetic stability, pluripotency, and differentiation capacity of iPSC lines under harmonized conditions [146]. In the context of drug safety, the Comprehensive in vitro Proarrhythmia Assay (CiPA) incorporates iPSC-CMs into multi-center studies to benchmark electrophysiological performance and evaluate proarrhythmic risk [147]. Together, these efforts help establish quality control benchmarks, reduce inter-laboratory variability, and expand the clinical and research utility of iPSC-based models.

### 5.3. Tumorigenicity and Genomic Instability

The pluripotent nature of iPSCs introduces potential risks of tumorigenesis, stemming from residual undifferentiated cells, sustained reprogramming activity, and genomic instability acquired during culture [148]. Advanced purification techniques [149], non-integrating reprogramming techniques [150,151,152], and ongoing assessments of genomic stability are critical to mitigating these risks and ensuring safety for clinical applications.

### 5.4. Immunogenicity and Compatibility Issues

Although autologous iPSCs are expected to minimize immune rejection, practical limitations in generating autologous therapies have led to the exploration of allogeneic approaches. These approaches carry the risk of immune responses due to genetic and epigenetic differences. Future research must focus on refining protocols to reduce immunogenicity while maintaining clinical feasibility [153].

### 5.5. Cell Engraftment and Integration Challenges

Achieving stable engraftment and functional integration of iPSC-derived cells into the host myocardium remains a major challenge. Poor survival, limited electromechanical coupling, and incomplete syncytium integration have hindered therapeutic success [154,155,156]. Approaches such as tissue-engineered scaffolds, improved delivery techniques, and the identification of paracrine mediators offer promising pathways to enhance therapeutic outcomes [157].

### 5.6. Cost, Expertise, and Ethical Considerations

The generation, maintenance, and differentiation of iPSCs require substantial financial resources and specialized expertise, which can limit accessibility. Streamlining processes through innovations in automation and artificial intelligence (AI)-driven platforms may reduce costs and improve large-scale feasibility, making iPSC-based solutions more feasible on a broader scale. Further advancements in AI-integrated protocols could optimize cell differentiation and quality control, accelerating the development process. 

Additionally, while iPSCs bypass many ethical concerns associated with embryonic stem cells, challenges remain regarding donor cell sourcing, clinical-grade production, and regulatory compliance. Initiatives such as Japan’s iPSC library [158] highlight potential solutions, but logistical and financial barriers persist.

Emerging ethical concerns related to iPSC use in CHD research and therapy also warrant attention. These include the responsible sourcing and consent for human-derived cells [159], equitable access to iPSC-based therapies [160], and the implications of gene editing technologies in pediatric populations [159,161]. As clinical applications advance, it is essential to establish transparent ethical frameworks that balance innovation with patient safety, informed consent, and long-term monitoring of interventions [162].

Moreover, the integration of clinically applicable imaging modalities, such as speckle tracking echocardiography (STE), can enhance the translational impact of iPSC-based studies. As an angle-independent technique increasingly used in both fetal and pediatric cardiology [163,164], STE enables detailed and reproducible assessment of global and regional myocardial function. In preclinical models, STE has also been used to evaluate the functional effects of iPSC-derived cell therapies on myocardial strain and remodeling [165]. Further studies are needed to explore its role in assessing the impact of iPSC-based interventions in congenital heart disease, particularly in evaluating regional and global myocardial responses.

## 6. Conclusions

Induced pluripotent stem cells have emerged as a transformative platform in CHD research and treatment. These technologies offer unparalleled opportunities to address critical gaps in understanding disease mechanisms, enabling innovative approaches to drug discovery, disease modeling, and regenerative therapies. Patient-specific iPSC models replicate the genetic and phenotypic heterogeneity of CHD, providing a foundation for precision medicine and targeted therapeutic interventions. Despite significant advancements, challenges such as the immaturity of iPSC-derived cells, variability in differentiation protocols, large-scale feasibility, and concerns about tumorigenicity remain barriers to clinical translation. To overcome these challenges, the development of standardized protocols, advanced bioengineering techniques, and rigorous quality control measures is crucial. Moreover, efforts to streamline costs, improve accessibility, and ensure regulatory compliance will be vital for widespread clinical adoption of iPSC-based therapies.

Looking ahead, the integration of cutting-edge technologies promises to further accelerate advancements in iPSC research. AI-driven approaches, such as predictive modeling and machine learning algorithms, are increasingly being applied to analyze large-scale iPSC data. These tools can enhance our understanding of how complex genetic and environmental factors influence CHD phenotypes, leading to more accurate disease prediction and personalized treatment strategies. Additionally, combining AI with high-throughput screening platforms may significantly optimize drug discovery, accelerating the identification of effective compounds tailored to patient-specific needs.

To fully realize the translational potential of iPSC-based technologies in CHD, strategic interdisciplinary collaboration is essential. Fields such as bioinformatics and computational biology are critical for analyzing genomic, transcriptomic, and electrophysiological data generated from iPSC-derived cardiac models. Materials science and tissue engineering contribute to the development of biomimetic scaffolds, 3D cardiac tissues, and organoids that better mimic physiological conditions. In parallel, clinical pharmacology and regulatory science support the optimization of dosing strategies and ensure safety and compliance throughout the translational process. By integrating expertise from these diverse disciplines, the field can accelerate discovery, improve model accuracy, and overcome key translational barriers in iPSC-based CHD research.

The continued evolution of iPSC research holds the promise of reshaping CHD care and advancing regenerative medicine. By fostering interdisciplinary collaboration among scientists, clinicians, and industry experts, the field can drive innovation toward patient-centered healthcare solutions. With sustained investment in research and a commitment to addressing existing challenges, iPSC technology is set to play a significant role in next-generation healthcare, offering renewed hope to patients and families affected by CHD worldwide.

## Data Availability

Not applicable.

## Abbreviations

The following abbreviations are used in this manuscript:

AI	Artificial intelligence
APC	Automated patch clamp
ASDs	Atrial septal defects
AVSD	Atrioventricular septal defect
BAV	Bicuspid aortic valve
BTHS	Barth syndrome
CAVD	Calcific aortic valve disease
cGMP	Current good manufacturing practices
CHD	Congenital heart disease
CiPA	Comprehensive in vitro Proarrhythmia Assay
CPC	Cardiac progenitor cell
CRISPR/Cas9	Clustered regularly interspaced short palindromic repeats/CRISPR-associated protein 9
DEGs	Differentially expressed genes
FDA	U.S. Food and Drug Administration
HLHS	Hypoplastic left heart syndrome
HOS	Holt–Oram syndrome
iPSCs	Induced pluripotent stem cells
iPSC-CMs	iPSC-derived cardiomyocytes
ISCI	International Stem Cell Initiative
LQTS	Long QT syndrome
LVNC	Left ventricular non-compaction
MEAs	Microelectrode arrays
miRNAs	MicroRNAs
MPS	Microphysiological systems
NIH	National Institutes of Health
OFT	Outflow tract
PAIVS	Pulmonary atresia with intact ventricular septum
PPAR	Peroxisome proliferator-activated receptor
ROS	Reactive oxygen species
scRNA-seq	Single-cell RNA sequencing
STE	Speckle tracking echocardiography
SVAS	Supravalvular aortic stenosis
SVD	Single ventricle defects
TOF	Tetralogy of Fallot
VSD	Ventricular septal defect

## References

[1] Hoffman J.I., Kaplan S. (2002). The incidence of congenital heart disease. J. Am. Coll. Cardiol..

[2] Bouma B.J., Mulder B.J. (2017). Changing landscape of congenital heart disease. Circ. Res..

[3] Cunningham R.M., Walton M.A., Carter P.M. (2018). The major causes of death in children and adolescents in the united states. N. Engl. J. Med..

[4] Hunt S., Baker D., Chin M., Cinquegrani M., Feldman A., Francis G. (2001). Heart failure society of america acc/aha guidelines for the evaluation and management of chronic heart failure in the adult: Executive summary. A report of the american college of cardiology/american heart association task force on practice guidelines (committee to revise the 1995 guidelines for the evaluation and management of heart failure): Developed in collaboration with the international society for heart and lung transplantation; endorsed by the heart failure society of america. Circulation.

[5] Jha B.S., Farnoodian M., Bharti K. (2021). Regulatory considerations for developing a phase i investigational new drug application for autologous induced pluripotent stem cells-based therapy product. Stem Cells Transl. Med..

[6] Balafkan N., Mostafavi S., Schubert M., Siller R., Liang K.X., Sullivan G., Bindoff L.A. (2020). A method for differentiating human induced pluripotent stem cells toward functional cardiomyocytes in 96-well microplates. Sci. Rep..

[7] Takahashi K., Yamanaka S. (2006). Induction of pluripotent stem cells from mouse embryonic and adult fibroblast cultures by defined factors. Cell.

[8] Takahashi K., Yamanaka S. (2016). A decade of transcription factor-mediated reprogramming to pluripotency. Nat. Rev. Mol. Cell Biol..

[9] Robertson J.A. (2001). Human embryonic stem cell research: Ethical and legal issues. Nat. Rev. Genet..

[10] Wert G.D., Mummery C. (2003). Human embryonic stem cells: Research, ethics and policy. Hum. Reprod..

[11] Lu J., Kong X., Luo C., Kathy Li K. (2013). Application of epigenome-modifying small molecules in induced pluripotent stem cells. Med. Res. Rev..

[12] Burridge P.W., Keller G., Gold J.D., Wu J.C. (2012). Production of de novo cardiomyocytes: Human pluripotent stem cell differentiation and direct reprogramming. Cell Stem Cell.

[13] Burridge P.W., Matsa E., Shukla P., Lin Z.C., Churko J.M., Ebert A.D., Lan F., Diecke S., Huber B., Mordwinkin N.M. (2014). Chemically defined generation of human cardiomyocytes. Nat. Methods.

[14] Gelb B.D., Chung W.K. (2014). Complex genetics and the etiology of human congenital heart disease. Cold Spring Harb. Perspect. Med..

[15] Buja L.M., Butany J. (2022). Cardiovascular Pathology.

[16] Kalisch-Smith J.I., Ved N., Sparrow D.B. (2020). Environmental risk factors for congenital heart disease. Cold Spring Harb. Perspect. Biol..

[17] Grunert M., Appelt S., Schönhals S., Mika K., Cui H., Cooper A., Cyganek L., Guan K., Sperling S.R. (2020). Induced pluripotent stem cells of patients with tetralogy of fallot reveal transcriptional alterations in cardiomyocyte differentiation. Sci. Rep..

[18] Kitani T., Tian L., Zhang T., Itzhaki I., Zhang J.Z., Ma N., Liu C., Rhee J.-W., Romfh A.W., Lui G.K. (2020). Rna sequencing analysis of induced pluripotent stem cell-derived cardiomyocytes from congenital heart disease patients. Circ. Res..

[19] Kim M.-S., Fleres B., Lovett J., Anfinson M., Samudrala S.S.K., Kelly L.J., Teigen L.E., Cavanaugh M., Marquez M., Geurts A.M. (2020). Contractility of induced pluripotent stem cell-cardiomyocytes with an myh6 head domain variant associated with hypoplastic left heart syndrome. Front. Cell Dev. Biol..

[20] Xu X., Zou R., Liu X., Su Q. (2022). Alternative splicing signatures of congenital heart disease and induced pluripotent stem cell-derived cardiomyocytes from congenital heart disease patients. Medicine.

[21] Xu X., Jin K., Bais A.S., Zhu W., Yagi H., Feinstein T.N., Nguyen P.K., Criscione J.D., Liu X., Beutner G. (2022). Uncompensated mitochondrial oxidative stress underlies heart failure in an ipsc-derived model of congenital heart disease. Cell Stem Cell.

[22] Yang C., Xu Y., Yu M., Lee D., Alharti S., Hellen N., Ahmad Shaik N., Banaganapalli B., Sheikh Ali Mohamoud H., Elango R. (2017). Induced pluripotent stem cell modelling of hlhs underlines the contribution of dysfunctional notch signalling to impaired cardiogenesis. Hum. Mol. Genet..

[23] Miao Y., Tian L., Martin M., Paige S.L., Galdos F.X., Li J., Klein A., Zhang H., Ma N., Wei Y. (2020). Intrinsic endocardial defects contribute to hypoplastic left heart syndrome. Cell Stem Cell.

[24] Collins F.S., Varmus H. (2015). A new initiative on precision medicine. N. Engl. J. Med..

[25] Navarrete E.G., Liang P., Lan F., Sanchez-Freire V., Simmons C., Gong T., Sharma A., Burridge P.W., Patlolla B., Lee A.S. (2013). Screening drug-induced arrhythmia using human induced pluripotent stem cell–derived cardiomyocytes and low-impedance microelectrode arrays. Circulation.

[26] Margulis M., Sorota S. (2008). Additive effects of combined application of multiple herg blockers. J. Cardiovasc. Pharmacol..

[27] Mantakaki A., Fakoya A.O.J., Sharifpanah F. (2018). Recent advances and challenges on application of tissue engineering for treatment of congenital heart disease. PeerJ.

[28] Majumdar U., Yasuhara J., Garg V. (2021). In vivo and in vitro genetic models of congenital heart disease. Cold Spring Harb. Perspect. Biol..

[29] Sanchez-Freire V., Lee A.S., Hu S., Abilez O.J., Liang P., Lan F., Huber B.C., Ong S.-G., Hong W.X., Huang M. (2014). Effect of human donor cell source on differentiation and function of cardiac induced pluripotent stem cells. J. Am. Coll. Cardiol..

[30] Hrstka S.C., Li X., Nelson T.J., Group W.P.G.P. (2017). Notch1-dependent nitric oxide signaling deficiency in hypoplastic left heart syndrome revealed through patient-specific phenotypes detected in bioengineered cardiogenesis. Stem Cells.

[31] Hall B., Alonzo M., Texter K., Garg V., Zhao M.T. (2022). Probing single ventricle heart defects with patient-derived induced pluripotent stem cells and emerging technologies. Birth Defects Res..

[32] Adhicary S., Ye S., Lin H., Texter K., Garg V., Zhao M.-T. (2023). Establishment of nchi009-a, an ipsc line from a patient with hypoplastic left heart syndrome (hlhs) carrying a heterozygous notch1 mutation. Stem Cell Res..

[33] Page D.J., Miossec M.J., Williams S.G., Monaghan R.M., Fotiou E., Cordell H.J., Sutcliffe L., Topf A., Bourgey M., Bourque G. (2019). Whole exome sequencing reveals the major genetic contributors to nonsyndromic tetralogy of fallot. Circ. Res..

[34] Contreras J., Alonzo M., Ye S., Lin H., Hernandez-Rosario L., McBride K.L., Texter K., Garg V., Zhao M.-T. (2022). Generation of an induced pluripotent stem cell line nchi003-a from a 11-year-old male with pulmonary atresia with intact ventricular septum (pa-ivs). Stem Cell Res..

[35] Hanley M., Alonzo M., Ye S., Yu Y., Contreras J., Hayden J., Garg V., Zhao M.-T. (2024). Characterization of an induced pluripotent stem cell line (nchi013-a) from a 5-year-old male with pulmonary atresia with intact ventricular septum and a biventricular repair. Stem Cell Res..

[36] Theodoris C.V., Li M., White M.P., Liu L., He D., Pollard K.S., Bruneau B.G., Srivastava D. (2015). Human disease modeling reveals integrated transcriptional and epigenetic mechanisms of notch1 haploinsufficiency. Cell.

[37] Ge X., Ren Y., Bartulos O., Lee M.Y., Yue Z., Kim K.-Y., Li W., Amos P.J., Bozkulak E.C., Iyer A. (2012). Modeling supravalvular aortic stenosis syndrome with human induced pluripotent stem cells. Circulation.

[38] Ang Y.-S., Rivas R.N., Ribeiro A.J., Srivas R., Rivera J., Stone N.R., Pratt K., Mohamed T.M., Fu J.-D., Spencer C.I. (2016). Disease model of gata4 mutation reveals transcription factor cooperativity in human cardiogenesis. Cell.

[39] Wang G., McCain M.L., Yang L., He A., Pasqualini F.S., Agarwal A., Yuan H., Jiang D., Zhang D., Zangi L. (2014). Modeling the mitochondrial cardiomyopathy of barth syndrome with induced pluripotent stem cell and heart-on-chip technologies. Nat. Med..

[40] Jaffré F. (2023). HiPSCs as a unique platform to model cardiogenesis in notch1-associated hlhs: HiPSCs to model complex congenital heart defects. Circ. Res..

[41] Ergir E., Oliver-De La Cruz J., Fernandes S., Cassani M., Niro F., Pereira-Sousa D., Vrbský J., Vinarský V., Perestrelo A.R., Debellis D. (2022). Generation and maturation of human ipsc-derived 3d organotypic cardiac microtissues in long-term culture. Sci. Rep..

[42] Simunovic M., Brivanlou A.H. (2017). Embryoids, organoids and gastruloids: New approaches to understanding embryogenesis. Development.

[43] Sumbal J., Chiche A., Charifou E., Koledova Z., Li H. (2020). Primary mammary organoid model of lactation and involution. Front. Cell Dev. Biol..

[44] Shimonosono M., Morimoto M., Hirose W., Tomita Y., Matsuura N., Flashner S., Ebadi M.S., Okayasu E.H., Lee C.Y., Britton W.R. (2024). Modeling epithelial homeostasis and perturbation in three-dimensional human esophageal organoids. Biomolecules.

[45] Volmert B., Kiselev A., Juhong A., Wang F., Riggs A., Kostina A., O’Hern C., Muniyandi P., Wasserman A., Huang A. (2023). A patterned human primitive heart organoid model generated by pluripotent stem cell self-organization. Nat. Commun..

[46] Ho B.X., Pang J.K.S., Chen Y., Loh Y.-H., An O., Yang H.H., Seshachalam V.P., Koh J.L., Chan W.-K., Ng S.Y. (2022). Robust generation of human-chambered cardiac organoids from pluripotent stem cells for improved modelling of cardiovascular diseases. Stem Cell Res. Ther..

[47] Hoang P., Kowalczewski A., Sun S., Winston T.S., Archilla A.M., Lemus S.M., Ercan-Sencicek A.G., Gupta A.R., Liu W., Kontaridis M.I. (2021). Engineering spatial-organized cardiac organoids for developmental toxicity testing. Stem Cell Rep..

[48] Cashman T.J., Josowitz R., Johnson B.V., Gelb B.D., Costa K.D. (2016). Human engineered cardiac tissues created using induced pluripotent stem cells reveal functional characteristics of braf-mediated hypertrophic cardiomyopathy. PLoS ONE.

[49] Lewis-Israeli Y.R., Wasserman A.H., Gabalski M.A., Volmert B.D., Ming Y., Ball K.A., Yang W., Zou J., Ni G., Pajares N. (2021). Self-assembling human heart organoids for the modeling of cardiac development and congenital heart disease. Nat. Commun..

[50] Siatra P., Vatsellas G., Chatzianastasiou A., Balafas E., Manolakou T., Papapetropoulos A., Agapaki A., Mouchtouri E.-T., Ruchaya P.J., Korovesi A.G. (2023). Return of the tbx5; lineage-tracing reveals ventricular cardiomyocyte-like precursors in the injured adult mammalian heart. NPJ Regen. Med..

[51] Schmidt C., Deyett A., Ilmer T., Haendeler S., Caballero A.T., Novatchkova M., Netzer M.A., Ginistrelli L.C., Juncosa E.M., Bhattacharya T. (2023). Multi-chamber cardioids unravel human heart development and cardiac defects. Cell.

[52] Lee J., Sutani A., Kaneko R., Takeuchi J., Sasano T., Kohda T., Ihara K., Takahashi K., Yamazoe M., Morio T. (2020). In vitro generation of functional murine heart organoids via fgf4 and extracellular matrix. Nat. Commun..

[53] Zhang M., Li F.X., Liu X.Y., Hou J.Y., Ni S.H., Wang J., Zhao C.M., Zhang W., Kong Y., Huang R.T. (2018). Tbx1 loss-of-function mutation contributes to congenital conotruncal defects. Exp. Ther. Med..

[54] Calmont A., Ivins S., Van Bueren K.L., Papangeli I., Kyriakopoulou V., Andrews W.D., Martin J.F., Moon A.M., Illingworth E.A., Basson M.A. (2009). Tbx1 controls cardiac neural crest cell migration during arch artery development by regulating gbx2 expression in the pharyngeal ectoderm. Development.

[55] Vitelli F., Morishima M., Taddei I., Lindsay E.A., Baldini A. (2002). Tbx1 mutation causes multiple cardiovascular defects and disrupts neural crest and cranial nerve migratory pathways. Hum. Mol. Genet..

[56] Phillips H.M., Stothard C.A., Shaikh Qureshi W.M., Kousa A.I., Briones-Leon J.A., Khasawneh R.R., O’Loughlin C., Sanders R., Mazzotta S., Dodds R. (2019). Pax9 is required for cardiovascular development and interacts with tbx1 in the pharyngeal endoderm to control 4th pharyngeal arch artery morphogenesis. Development.

[57] Khalil A., Tanos R., El-Hachem N., Kurban M., Bouvagnet P., Bitar F., Nemer G. (2017). A hand to tbx5 explains the link between thalidomide and cardiac diseases. Sci. Rep..

[58] Yang J., Lei W., Xiao Y., Tan S., Yang J., Lin Y., Yang Z., Zhao D., Zhang C., Shen Z. (2024). Generation of human vascularized and chambered cardiac organoids for cardiac disease modelling and drug evaluation. Cell Prolif..

[59] Hiroi Y., Kudoh S., Monzen K., Ikeda Y., Yazaki Y., Nagai R., Komuro I. (2001). Tbx5 associates with nkx2-5 and synergistically promotes cardiomyocyte differentiation. Nat. Genet..

[60] Friedman C.E., Nguyen Q., Lukowski S.W., Helfer A., Chiu H.S., Miklas J., Levy S., Suo S., Han J.-D.J., Osteil P. (2018). Single-cell transcriptomic analysis of cardiac differentiation from human pscs reveals hopx-dependent cardiomyocyte maturation. Cell Stem Cell.

[61] Liu Z., Wang L., Welch J.D., Ma H., Zhou Y., Vaseghi H.R., Yu S., Wall J.B., Alimohamadi S., Zheng M. (2017). Single-cell transcriptomics reconstructs fate conversion from fibroblast to cardiomyocyte. Nature.

[62] Cheng S., Brenière-Letuffe D., Ahola V., Wong A.O., Keung H.Y., Gurung B., Zheng Z., Costa K.D., Lieu D.K., Keung W. (2023). Single-cell rna sequencing reveals maturation trajectory in human pluripotent stem cell-derived cardiomyocytes in engineered tissues. iScience.

[63] Nguyen Q.H., Lukowski S.W., Chiu H.S., Friedman C.E., Senabouth A., Crowhurst L., Bruxmer T.J., Christ A.N., Palpant N.J., Powell J.E. (2017). Determining cell fate specification and genetic contribution to cardiac disease risk in hipsc-derived cardiomyocytes at single cell resolution. BioRxiv.

[64] Lam Y.Y., Keung W., Chan C.H., Geng L., Wong N., Brenière-Letuffe D., Li R.A., Cheung Y.F. (2020). Single-cell transcriptomics of engineered cardiac tissues from patient-specific induced pluripotent stem cell–derived cardiomyocytes reveals abnormal developmental trajectory and intrinsic contractile defects in hypoplastic right heart syndrome. J. Am. Heart Assoc..

[65] Han S., Xu Q., Du Y., Tang C., Cui H., Xia X., Zheng R., Sun Y., Shang H. (2024). Single-cell spatial transcriptomics in cardiovascular development, disease, and medicine. Genes Dis..

[66] Wan X., Xiao J., Tam S.S.T., Cai M., Sugimura R., Wang Y., Wan X., Lin Z., Wu A.R., Yang C. (2023). Integrating spatial and single-cell transcriptomics data using deep generative models with spatialscope. Nat. Commun..

[67] Palmer J.A., Rosenthal N., Teichmann S.A., Litvinukova M. (2024). Revisiting cardiac biology in the era of single cell and spatial omics. Circ. Res..

[68] Roth R., Kim S., Kim J., Rhee S. (2020). Single-cell and spatial transcriptomics approaches of cardiovascular development and disease. BMB Rep..

[69] Jiang Y., Habibollah S., Tilgner K., Collin J., Barta T., Al-Aama J.Y., Tesarov L., Hussain R., Trafford A.W., Kirkwood G. (2014). An induced pluripotent stem cell model of hypoplastic left heart syndrome (hlhs) reveals multiple expression and functional differences in hlhs-derived cardiac myocytes. Stem Cells Transl. Med..

[70] Paige S.L., Galdos F.X., Lee S., Chin E.T., Ranjbarvaziri S., Feyen D.A., Darsha A.K., Xu S., Ryan J.A., Beck A.L. (2020). Patient-specific induced pluripotent stem cells implicate intrinsic impaired contractility in hypoplastic left heart syndrome. Circulation.

[71] Kobayashi J., Yoshida M., Tarui S., Hirata M., Nagai Y., Kasahara S., Naruse K., Ito H., Sano S., Oh H. (2014). Directed differentiation of patient-specific induced pluripotent stem cells identifies the transcriptional repression and epigenetic modification of nkx2-5, hand1, and notch1 in hypoplastic left heart syndrome. PLoS ONE.

[72] Zhu J.-y., van de Leemput J., Han Z. (2023). The roles of histone lysine methyltransferases in heart development and disease. J. Cardiovasc. Dev. Dis..

[73] Davis K., Azarcon P., Hickenlooper S., Bia R., Horiuchi E., Szulik M.W., Franklin S. (2021). The role of demethylases in cardiac development and disease. J. Mol. Cell. Cardiol..

[74] Liu J., Kong S., Song S., Dong H., Zhang Z., Fan T. (2022). Metabolic variation dictates cardiac pathogenesis in patients with tetralogy of fallot. Front. Pediatr..

[75] Li Y., Tian M., Zhou Z., Tu J., Zhang R., Huang Y., Zhang Y., Cui H., Zhuang J., Chen J. (2025). Integrative metabolomics dictate distinctive signature profiles in patients with tetralogy of fallot. Pediatr. Res..

[76] Yang B., Zhou W., Jiao J., Nielsen J.B., Mathis M.R., Heydarpour M., Lettre G., Folkersen L., Prakash S., Schurmann C. (2017). Protein-altering and regulatory genetic variants near gata4 implicated in bicuspid aortic valve. Nat. Commun..

[77] Huang T., Cheng J., Feng H., Zhou W., Qiu P., Zhou D., Yang D., Zhang J., Willer C., Chen Y.E. (2023). Bicuspid aortic valve–associated regulatory regions reveal gata4 regulation and function during human-induced pluripotent stem cell–based endothelial-mesenchymal transition—Brief report. Arterioscler. Thromb. Vasc. Biol..

[78] Ye L., Yu Y., Zhao Z.-A., Zhao D., Ni X., Wang Y., Fang X., Yu M., Wang Y., Tang J.-M. (2022). Patient-specific ipsc-derived cardiomyocytes reveal abnormal regulation of fgf16 in a familial atrial septal defect. Cardiovasc. Res..

[79] Sharma A., Wasson L.K., Willcox J.A., Morton S.U., Gorham J.M., DeLaughter D.M., Neyazi M., Schmid M., Agarwal R., Jang M.Y. (2020). Gata6 mutations in hipscs inform mechanisms for maldevelopment of the heart, pancreas, and diaphragm. eLife.

[80] Zhou L., Liu J., Xiang M., Olson P., Guzzetta A., Zhang K., Moskowitz I.P., Xie L. (2017). Gata4 potentiates second heart field proliferation and hedgehog signaling for cardiac septation. Proc. Natl. Acad. Sci. USA.

[81] Itzhaki I., Maizels L., Huber I., Zwi-Dantsis L., Caspi O., Winterstern A., Feldman O., Gepstein A., Arbel G., Hammerman H. (2011). Modelling the long qt syndrome with induced pluripotent stem cells. Nature.

[82] Liang P., Sallam K., Wu H., Li Y., Itzhaki I., Garg P., Zhang Y., Termglichan V., Lan F., Gu M. (2016). Patient-specific and genome-edited induced pluripotent stem cell–derived cardiomyocytes elucidate single-cell phenotype of brugada syndrome. J. Am. Coll. Cardiol..

[83] McKeithan W.L., Savchenko A., Yu M.S., Cerignoli F., Bruyneel A.A., Price J.H., Colas A.R., Miller E.W., Cashman J.R., Mercola M. (2017). An automated platform for assessment of congenital and drug-induced arrhythmia with hipsc-derived cardiomyocytes. Front. Physiol..

[84] Gifford C.A., Ranade S.S., Samarakoon R., Salunga H.T., De Soysa T.Y., Huang Y., Zhou P., Elfenbein A., Wyman S.K., Bui Y.K. (2019). Oligogenic inheritance of a human heart disease involving a genetic modifier. Science.

[85] van Ouwerkerk A.F., Bosada F.M., van Duijvenboden K., Houweling A.C., Scholman K.T., Wakker V., Allaart C.P., Uhm J.-S., Mathijssen I.B., Baartscheer T. (2022). Patient-specific tbx5-g125r variant induces profound transcriptional deregulation and atrial dysfunction. Circulation.

[86] Lahm H., Dzilic E., Neb I., Doppler S.A., Schneider S., Lange R., Krane M., Dreßen M. (2023). Correction of a deleterious tbx5 mutation in an induced pluripotent stem cell line (dhmi004-a-1) using a completely plasmid-free crispr/cas 9 approach. Stem Cell Res..

[87] Jiang Y., Tarzami S., Burch J.B., Evans T. (1998). Common role for each of the cgata-4/5/6 genes in the regulation of cardiac morphogenesis. Dev. Genet..

[88] Franco E.D., Shaw-Smith C., Flanagan S.E., Shepherd M., Hattersley A.T., Ellard S. (2013). GATA6 mutations cause a broad phenotypic spectrum of diabetes from pancreatic agenesis to adult-onset diabetes without exocrine insufficiency. Diabetes.

[89] Richards A.A., Garg V. (2010). Genetics of congenital heart disease. Curr. Cardiol. Rev..

[90] Miles M.L., Cowan N., Jackson G. (2020). A nonsense gata6 mutation explains history of congenital heart defects and 10 years of poorly-controlled diabetes lacking dka in a non-obese 30 year-old incidentally found to have pancreatic hypoplasia. AACE Clin. Case Rep..

[91] Maitra M., Koenig S.N., Srivastava D., Garg V. (2010). Identification of gata6 sequence variants in patients with congenital heart defects. Pediatr. Res..

[92] Pugnaloni F., Martini L., De Rose D.U., Landolfo F., Giliberti P., Ruta R., Novelli A., Rapini N., Barbetti F., Toscano A. (2024). A new variant in the GATA6 gene associated with tracheoesophageal fistula, pulmonary vein stenosis and neonatal diabetes. Horm. Res. Paediatr..

[93] Homsy J., Zaidi S., Shen Y., Ware J.S., Samocha K.E., Karczewski K.J., DePalma S.R., McKean D., Wakimoto H., Gorham J. (2015). De novo mutations in congenital heart disease with neurodevelopmental and other congenital anomalies. Science.

[94] Zaidi S., Brueckner M. (2017). Genetics and genomics of congenital heart disease. Circ. Res..

[95] Sevim Bayrak C., Zhang P., Tristani-Firouzi M., Gelb B.D., Itan Y. (2020). De novo variants in exomes of congenital heart disease patients identify risk genes and pathways. Genome Med..

[96] Chai S., Wan X., Ramirez-Navarro A., Tesar P.J., Kaufman E.S., Ficker E., George A.L., Deschênes I. (2018). Physiological genomics identifies genetic modifiers of long qt syndrome type 2 severity. J. Clin. Investig..

[97] Zhang X., Wang C., He D., Cheng Y., Li Y., Qi D., Li B., Zheng F. (2022). Identification of DNA methylation-regulated genes as potential biomarkers for coronary heart disease via machine learning in the framingham heart study. Clin. Epigenetics.

[98] Chhatwal K., Smith J.J., Bola H., Zahid A., Venkatakrishnan A., Brand T. (2023). Uncovering the genetic basis of congenital heart disease: Recent advancements and implications for clinical management. CJC Pediatr. Congenit. Heart Dis..

[99] Kawaguchi S., Moukette B., Hayasaka T., Haskell A.K., Mah J., Sepúlveda M.N., Tang Y., Kim I.-m. (2023). Noncoding rnas as key regulators for cardiac development and cardiovascular diseases. J. Cardiovasc. Dev. Dis..

[100] Diez-Cuñado M., Wei K., Bushway P.J., Maurya M.R., Perera R., Subramaniam S., Ruiz-Lozano P., Mercola M. (2018). Mirnas that induce human cardiomyocyte proliferation converge on the hippo pathway. Cell Rep..

[101] Ashiq S., Sabar M.F. (2022). The role of genetics in the understanding of complex congenital heart diseases. Pak. Heart J..

[102] Blinova K., Dang Q., Millard D., Smith G., Pierson J., Guo L., Brock M., Lu H., Kraushaar U., Zeng H. (2018). International multisite study of human-induced pluripotent stem cell-derived cardiomyocytes for drug proarrhythmic potential assessment. Cell Rep..

[103] Blinova K., Stohlman J., Vicente J., Chan D., Johannesen L., Hortigon-Vinagre M.P., Zamora V., Smith G., Crumb W.J., Pang L. (2017). Comprehensive translational assessment of human-induced pluripotent stem cell derived cardiomyocytes for evaluating drug-induced arrhythmias. Toxicol. Sci..

[104] Kitani T., Ong S.-G., Lam C.K., Rhee J.-W., Zhang J.Z., Oikonomopoulos A., Ma N., Tian L., Lee J., Telli M.L. (2019). Human-induced pluripotent stem cell model of trastuzumab-induced cardiac dysfunction in patients with breast cancer. Circulation.

[105] Liang P., Lan F., Lee A.S., Gong T., Sanchez-Freire V., Wang Y., Diecke S., Sallam K., Knowles J.W., Wang P.J. (2013). Drug screening using a library of human induced pluripotent stem cell–derived cardiomyocytes reveals disease-specific patterns of cardiotoxicity. Circulation.

[106] Zhang H., Ren X., Wu C., He X., Huang Z., Li Y., Liao L., Xiang J., Li M., Wu L. (2024). Intracellular calcium dysregulation in heart and brain diseases: Insights from induced pluripotent stem cell studies. J. Neuropathol. Exp. Neurol..

[107] Yu Y., Deschenes I., Zhao M.-T. (2023). Precision medicine for long qt syndrome: Patient-specific ipscs take the lead. Expert Rev. Mol. Med..

[108] Lin H., McBride K.L., Garg V., Zhao M.-T. (2021). Decoding genetics of congenital heart disease using patient-derived induced pluripotent stem cells (ipscs). Front. Cell Dev. Biol..

[109] Garg P., Oikonomopoulos A., Chen H., Li Y., Lam C.K., Sallam K., Perez M., Lux R.L., Sanguinetti M.C., Wu J.C. (2018). Genome editing of induced pluripotent stem cells to decipher cardiac channelopathy variant. J. Am. Coll. Cardiol..

[110] Cheng Y.-Y., Hu Y.-F., Hsieh P.C.-H. (2023). The role of large animal models in cardiac regeneration research using human pluripotent stem cell-derived cardiomyocytes. Curr. Cardiol. Rep..

[111] Vo Q.D., Saito Y., Nakamura K., Iida T., Yuasa S. (2024). Induced pluripotent stem cell-derived cardiomyocytes therapy for ischemic heart disease in animal model: A meta-analysis. Int. J. Mol. Sci..

[112] Liu D., Chen M., Mendoza B., Cheng H., Hu R., Li L., Trinh C.T., Tuskan G.A., Yang X. (2019). Crispr/cas9-mediated targeted mutagenesis for functional genomics research of crassulacean acid metabolism plants. J. Exp. Bot..

[113] Hunter C.T. (2021). Crispr/cas9 targeted mutagenesis for functional genetics in maize. Plants.

[114] Zhao T., Bai R., Wu F., Lu W.-J., Zhang J. (2021). Generation of a tbx5 homozygous knockout embryonic stem cell line (wae009-a-45) by crispr/cas9 genome editing. Stem Cell Res..

[115] Hockemeyer D., Jaenisch R. (2016). Induced pluripotent stem cells meet genome editing. Cell Stem Cell.

[116] Deacon D.C., Happe C.L., Chen C., Tedeschi N., Manso A.M., Li T., Dalton N.D., Peng Q., Farah E.N., Gu Y. (2019). Combinatorial interactions of genetic variants in human cardiomyopathy. Nat. Biomed. Eng..

[117] Fear V.S., Forbes C.A., Shaw N.C., Farley K.O., Mantegna J.L., Htun J.P., Syn G., Viola H., Cserne Szappanos H., Hool L. (2023). Gene editing and cardiac disease modelling for the interpretation of genetic variants of uncertain significance in congenital heart disease. Stem Cell. Res. Ther..

[118] Carvalho T. (2023). Stem cell–derived heart cells injected into first patient. Nat. Med..

[119] HeartWorks I. (2023). “Autologous Induced Pluripotent Stem Cells of Cardiac Lineage for Congenital Heart Disease.” World Health Organization International Clinical Trials Registry Platform. https://clinicaltrials.gov/ct2/show/NCT05647213.

[120] U.S. FOOD & DRUG (2022). “Regulation of Human Cells, Tissues, and Cellular and Tissue-Based Products (hct/ps) Small Entity Compliance Guide.” Silver Spring, MD: Center for Biologics Evaluation and Research, U.S. Department of Health and Human Services. https://www.fda.gov/media/70689/download.

[121] Martin U. (2017). Therapeutic application of pluripotent stem cells: Challenges and risks. Front. Med..

[122] Mason C., Dunnill P. (2008). A brief definition of regenerative medicine. Regen. Med..

[123] Del Álamo J.C., Lemons D., Serrano R., Savchenko A., Cerignoli F., Bodmer R., Mercola M. (2016). High throughput physiological screening of ipsc-derived cardiomyocytes for drug development. Biochim. Biophys. Acta, Mol. Cell Res..

[124] Danker T., Möller C. (2014). Early identification of herg liability in drug discovery programs by automated patch clamp. Front. Pharmacol..

[125] Willems E., Kimler K., Boucher S., Asprer J., Sylakowski K., Lakshmipathy U., Kuninger D., Piper D. A robust platform for generation and high throughput functional analysis of human ipsc-derived cardiomyocytes. Proceedings of the Drug Discovery (ACC 2016).

[126] Feyen D.A., Perea-Gil I., Maas R.G., Harakalova M., Gavidia A.A., Arthur Ataam J., Wu T.-H., Vink A., Pei J., Vadgama N. (2021). Unfolded protein response as a compensatory mechanism and potential therapeutic target in pln r14del cardiomyopathy. Circulation.

[127] Mathur A., Loskill P., Shao K., Huebsch N., Hong S., Marcus S.G., Marks N., Mandegar M., Conklin B.R., Lee L.P. (2015). Human ipsc-based cardiac microphysiological system for drug screening applications. Sci. Rep..

[128] Fermini B., Hancox J.C., Abi-Gerges N., Bridgland-Taylor M., Chaudhary K.W., Colatsky T., Correll K., Crumb W., Damiano B., Erdemli G. (2016). A new perspective in the field of cardiac safety testing through the comprehensive in vitro proarrhythmia assay paradigm. J. Biomol. Screen..

[129] Qu Y., Gao B., Fang M., Vargas H.M. (2013). Human embryonic stem cell derived cardiac myocytes detect herg-mediated repolarization effects, but not nav1. 5 induced depolarization delay. J. Pharmacol. Toxicol. Methods.

[130] Bruyneel A.A., Muser T., Parthasarathy V., Feyen D., Mercola M., Serpooshan V., Wu S.M. (2019). Phenotypic screening of ipsc-derived cardiomyocytes for cardiotoxicity testing and therapeutic target discovery. Cardiovascular Regenerative Medicine: Tissue Engineering and Clinical Applications.

[131] Lian X., Hsiao C., Wilson G., Zhu K., Hazeltine L.B., Azarin S.M., Raval K.K., Zhang J., Kamp T.J., Palecek S.P. (2012). Robust cardiomyocyte differentiation from human pluripotent stem cells via temporal modulation of canonical wnt signaling. Proc. Natl. Acad. Sci. USA.

[132] Kempf H., Olmer R., Kropp C., Rückert M., Jara-Avaca M., Robles-Diaz D., Franke A., Elliott D.A., Wojciechowski D., Fischer M. (2014). Controlling expansion and cardiomyogenic differentiation of human pluripotent stem cells in scalable suspension culture. Stem Cell Rep..

[133] Ronaldson-Bouchard K., Vunjak-Novakovic G. (2018). Organs-on-a-chip: A fast track for engineered human tissues in drug development. Cell Stem Cell.

[134] Zhang F., Qiu H., Dong X., Zhang X., Wang C., Li X., Zhang X., Na J., Zhou J., Wang C. (2022). Single-cell atlas of multilineage cardiac organoids derived from human induced pluripotent stem cells. Life Med..

[135] Cao M., Liu Y., Sun Y., Han R., Jiang H. (2025). Current advances in human-induced pluripotent stem cell-based models and therapeutic approaches for congenital heart disease. Mol. Cell. Biochem..

[136] Karbassi E., Fenix A., Marchiano S., Muraoka N., Nakamura K., Yang X., Murry C.E. (2020). Cardiomyocyte maturation: Advances in knowledge and implications for regenerative medicine. Nat. Rev. Cardiol..

[137] Parikh S.S., Blackwell D.J., Gomez-Hurtado N., Frisk M., Wang L., Kim K., Dahl C.P., Fiane A., Tønnessen T., Kryshtal D.O. (2017). Thyroid and glucocorticoid hormones promote functional t-tubule development in human-induced pluripotent stem cell–derived cardiomyocytes. Circ. Res..

[138] Ronaldson-Bouchard K., Ma S.P., Yeager K., Chen T., Song L., Sirabella D., Morikawa K., Teles D., Yazawa M., Vunjak-Novakovic G. (2018). Advanced maturation of human cardiac tissue grown from pluripotent stem cells. Nature.

[139] Giacomelli E., Meraviglia V., Campostrini G., Cochrane A., Cao X., Van Helden R.W., Garcia A.K., Mircea M., Kostidis S., Davis R.P. (2020). Human-ipsc-derived cardiac stromal cells enhance maturation in 3d cardiac microtissues and reveal non-cardiomyocyte contributions to heart disease. Cell Stem Cell.

[140] Rossi G., Manfrin A., Lutolf M.P. (2018). Progress and potential in organoid research. Nat. Rev. Genet..

[141] Panopoulos A.D., Ruiz S., Yi F., Herrerias A., Batchelder E.M., Belmonte J.C.I. (2011). Rapid and highly efficient generation of induced pluripotent stem cells from human umbilical vein endothelial cells. PLoS ONE.

[142] Rowe R.G., Daley G.Q. (2019). Induced pluripotent stem cells in disease modelling and drug discovery. Nat. Rev. Genet..

[143] Bellin M., Casini S., Davis R.P., D’aniello C., Haas J., Ward-van Oostwaard D., Tertoolen L.G., Jung C.B., Elliott D.A., Welling A. (2013). Isogenic human pluripotent stem cell pairs reveal the role of a kcnh2 mutation in long-qt syndrome. EMBO J..

[144] Avior Y., Sagi I., Benvenisty N. (2016). Pluripotent stem cells in disease modelling and drug discovery. Nat. Rev. Mol. Cell Biol..

[145] Soares F.A., Sheldon M., Rao M., Mummery C., Vallier L. (2014). International coordination of large-scale human induced pluripotent stem cell initiatives: Wellcome trust and isscr workshops white paper. Stem Cell Rep..

[146] Kim J.-H., Kurtz A., Yuan B.-Z., Zeng F., Lomax G., Loring J.F., Crook J., Ju J.H., Clarke L., Inamdar M.S. (2017). Report of the international stem cell banking initiative workshop activity: Current hurdles and progress in seed-stock banking of human pluripotent stem cells. Stem Cells Transl. Med..

[147] Colatsky T., Fermini B., Gintant G., Pierson J.B., Sager P., Sekino Y., Strauss D.G., Stockbridge N. (2016). The comprehensive in vitro proarrhythmia assay (cipa) initiative—Update on progress. J. Pharmacol. Toxicol. Methods.

[148] Yamanaka S. (2020). Pluripotent stem cell-based cell therapy—Promise and challenges. Cell Stem Cell.

[149] Hayashi R., Ishikawa Y., Katori R., Sasamoto Y., Taniwaki Y., Takayanagi H., Tsujikawa M., Sekiguchi K., Quantock A.J., Nishida K. (2017). Coordinated generation of multiple ocular-like cell lineages and fabrication of functional corneal epithelial cell sheets from human ips cells. Nat. Protoc..

[150] Tavernier G., Wolfrum K., Demeester J., De Smedt S.C., Adjaye J., Rejman J. (2012). Activation of pluripotency-associated genes in mouse embryonic fibroblasts by non-viral transfection with in vitro-derived mrnas encoding oct4, sox2, klf4 and cmyc. Biomaterials.

[151] Kim D., Kim C.-H., Moon J.-I., Chung Y.-G., Chang M.-Y., Han B.-S., Ko S., Yang E., Cha K.Y., Lanza R. (2009). Generation of human induced pluripotent stem cells by direct delivery of reprogramming proteins. Cell Stem Cell.

[152] Chen X., Lu Y., Wang L., Ma X., Pu J., Lin L., Deng Q., Li Y., Wang W., Jin Y. (2023). A fast chemical reprogramming system promotes cell identity transition through a diapause-like state. Nat. Cell Biol..

[153] Zhao T., Zhang Z.-N., Rong Z., Xu Y. (2011). Immunogenicity of induced pluripotent stem cells. Nature.

[154] Hendry S.L., van der Bogt K.E., Sheikh A.Y., Arai T., Dylla S.J., Drukker M., McConnell M.V., Kutschka I., Hoyt G., Cao F. (2008). Multimodal evaluation of in vivo magnetic resonance imaging of myocardial restoration by mouse embryonic stem cells. J. Thorac. Cardiovasc. Surg..

[155] Hung T.-C., Suzuki Y., Urashima T., Caffarelli A., Hoyt G., Sheikh A.Y., Yeung A.C., Weissman I., Robbins R.C., Bulte J.M. (2008). Multimodality evaluation of the viability of stem cells delivered into different zones of myocardial infarction. Circ. Cardiovasc. Imaging.

[156] Terrovitis J., Stuber M., Youssef A., Preece S., Leppo M., Kizana E., Schär M., Gerstenblith G., Weiss R.G., Marbán E. (2008). Magnetic resonance imaging overestimates ferumoxide-labeled stem cell survival after transplantation in the heart. Circulation.

[157] Sanganalmath S.K., Bolli R. (2013). Cell therapy for heart failure: A comprehensive overview of experimental and clinical studies, current challenges, and future directions. Circ. Res..

[158] Turner M., Leslie S., Martin N.G., Peschanski M., Rao M., Taylor C.J., Trounson A., Turner D., Yamanaka S., Wilmut I. (2013). Toward the development of a global induced pluripotent stem cell library. Cell Stem Cell.

[159] King N.M., Perrin J. (2014). Ethical issues in stem cell research and therapy. Stem Cell. Res. Ther..

[160] Yap K.K. (2016). Inequality issues in stem cell medicine. Stem Cells Transl. Med..

[161] Baylis F., Robert J.S. (2004). The inevitability of genetic enhancement technologies. Bioethics.

[162] Sugarman J. (2008). Human stem cell ethics: Beyond the embryo. Cell Stem Cell.

[163] Li L., Sun J.P., Zuo R., Shen Y., Zhao M., Zhao W., Luo Z. (2023). Cardiac function evaluated by two-dimensional speckle tracking imaging in fetuses with congenital heart disease of ventricular afterload increase. J. Matern. Fetal Neonatal Med..

[164] Sonaglioni A., Bruno A., Nicolosi G.L., Bianchi S., Lombardo M., Muti P. (2024). Echocardiographic assessment of biventricular mechanics of fetuses and infants of gestational diabetic mothers: A systematic review and meta-analysis. Children.

[165] Miao Q., Shim W., Tee N., Lim S.Y., Chung Y.Y., Ja K.M.M., Ooi T.H., Tan G., Kong G., Wei H. (2014). Ipsc-derived human mesenchymal stem cells improve myocardial strain of infarcted myocardium. J. Cell. Mol. Med..

